# Preparation and Characterization of Isosorbide-Based Self-Healable Polyurethane Elastomers with Thermally Reversible Bonds

**DOI:** 10.3390/molecules24061061

**Published:** 2019-03-18

**Authors:** Han-Na Kim, Dae-Woo Lee, Hoon Ryu, Gwang-Seok Song, Dai-Soo Lee

**Affiliations:** 1 Division of Semiconductor and Chemical Engineering, Chonbuk National University, 567 Baekjedaero, Deokjin-gu, Jeonju 54896, Korea; hnk07@hanmail.net (H.-N.K.); dwlee2310@hanmail.net (D.-W.L.); 2 Industrial Biotechnology Program, Chemical R&D Center, Samyang Corporation, Daedeok-daero 730, Yuseong-gu, Daejeon 34055, Korea; hoon.ryu@samyang.com (H.R.); gwangseok.song@samyang.com (G.-S.S.)

**Keywords:** isosorbide, isomannide, thermo-reversible, self-healable, bio-based polyurethane

## Abstract

Polyurethane (PU) is a versatile polymer used in a wide range of applications. Recently, imparting PU with self-healing properties has attracted much interest to improve the product durability. The self-healing mechanism conceivably occurs through the existence of dynamic reversible bonds over a specific temperature range. The present study investigates the self-healing properties of 1,4:3,6-dianhydrohexitol-based PUs prepared from a prepolymer of poly(tetra-methylene ether glycol) and 4,4′-methylenebis(phenyl isocyanate) with different chain extenders (isosorbide or isomannide). PU with the conventional chain extender 1,4-butanediol was prepared for comparison. The urethane bonds in 1,4:3,6-dianhydrohexitol-based PUs were thermally reversible (as confirmed by the generation of isocyanate peaks observed by Fourier transform infrared spectroscopy) at mildly elevated temperatures and the PUs showed good mechanical properties. Especially the isosorbide-based polyurethane showed potential self-healing ability under mild heat treatment, as observed in reprocessing tests. It is inferred that isosorbide, bio-based bicyclic diol, can be employed as an efficient chain extender of polyurethane prepolymers to improve self-healing properties of polyurethane elastomers via reversible features of the urethane bonds.

## 1. Introduction

Polyurethane (PU) is a very versatile material with a wide range of physical and chemical properties. Consequently, it is widely applied in the automotive, construction, furniture, insulation, and textile industries [1]. Polyurethane elastomer (PUE), a linear block copolymer composed of a soft and a hard segment, is especially versatile. The soft segment (polyol) imparts the elastic properties of the polymer, and the hard segment (diisocyanate with a chain extender) acts like a physical crosslinker [2]. The strength and stiffness of the segmented structure can be controlled by varying the constituents of these three materials (polyol, diisocyanate, and chain extender) [3,4,5]. From an economic viability and safety perspective, other researchers have attempted to increase the lifetime of PU products [6,7]. Recently, the introduction of dynamic covalent bonds for self-healing polymers has attracted increasing interest. One self-healing mechanism involves reversible covalent bonding between the isocyanate and active hydrogens in PUs [8,9]. As the bond between isocyanate and aromatic hydroxyl compounds is relatively weak, it dissociates at a certain temperature, releasing free isocyanate and an aromatic hydroxyl. The dissociation temperature of thermo-reversible urethane bonds depends on the structures of the isocyanate and active hydrogen compound [10,11]. The reversible properties were utilized for the development of blocked isocyanates. Under appropriate temperature control, these covalent bonds can be repeatedly formed and dissociated, enabling self-healing in even thermosetting PUs [12,13,14].

In recent years, the replacement of petroleum-based materials with greener alternatives has become an urgent topic in both academia and industry, as petroleum resources are depleting and their disposal causes environmental problems [15,16,17]. As candidates among renewable resources, carbohydrate derivatives are highly suitable because they are biodegradable, biocompatible, low cost, and naturally abundant [18,19]. 1,4:3,6-Dianhydrohexitol exists in three isomeric forms with different chiralities: isosorbide (1,4:3,6-dianhydro-d-glucitol), isomannide (1,4:3,6-dianhydro-d-mannitol), and isoidide (1,4:3,6-dianhydro-l-idotol) [20]. 1,4:3,6-Dianhydrohexitol is a biobased bicyclic diol consisting of two *cis*-shaped tetrahydrofuran rings and two hydroxyl groups, with an internal angle of approximately 120°. Isosorbide has one highly reactive hydroxyl group at the exo-position (OH-2) and one relatively less reactive hydroxyl group at the endo-position (OH-5). At the endo-position, the reactivity is hindered by steric effects and hydrogen bonds with nearby ether groups. Isomannide has two endo-hydroxyl groups, whereas isoidide has two exo-hydroxyl groups [21,22]. Polymers based on 1,4:3,6-dianhydrohexitol have high glass transition temperatures [23,24] and special optical properties [25], and are applied as food packaging materials [26] and medical fields [27,28,29]. An PU based on isosorbide was first reported in 1984 [30]. More recently, isosorbide [31,32,33,34] and isomannide [35] have been studied to replace the petrochemical-based chain extender (1,4-butanediol) being used in conventional PU manufacture. To the best of our knowledge, the thermally reversible nature of urethane bonds formed by the chemical reactions between 1,4:3,6-dianhydrohexitol and aromatic diisocyanates was not investigated up to now.

In this paper, we investigated PUEs based on isosorbide and isomannide, and study the applicability of the reversible properties of the urethane bonds to self-healing polymers using the structural features of 1,4:3,6-dianhydrohexitol. We prepared a prepolymer by reacting polyol with an aromatic diisocyanate. We then produced PUEs using butane diol, isosorbide, or isomannide as the chain extender. The urethane groups formed from isosorbide and isomannide display reversible properties at lower temperature than 1,4-butanediol based urethane groups, allowing reprocessing and thermally self-healing properties at mild temperatures. 

## 2. Results and Discussion

### 2.1. Synthesis and Characterization of PUEs

**Scheme 1:**
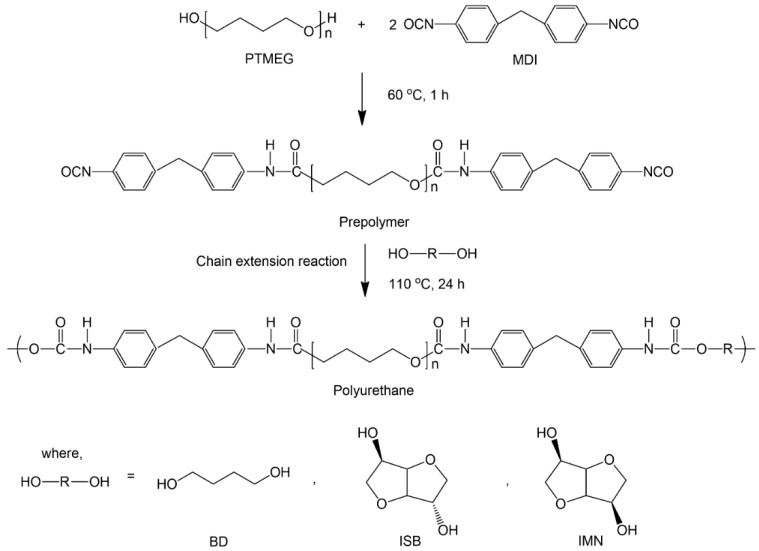
Synthesis of PUEs from prepolymer with different chain extenders.

**Figure 1:**
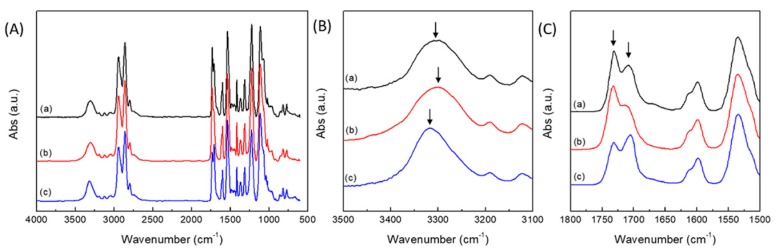
FT-IR spectra of PUEs in the whole range of wave numbers (**A**) and around specific wave numbers (**B**,**C**) in expansion: (a) BD–PU, (b) ISB–PU, and (c) IMN–PU.

The process to prepare PUEs is described in Scheme 1. The successful synthesis of the prepared PUEs was confirmed by FT-IR spectroscopy. Figure 1 shows the FT-IR spectra of BD–PU, ISB–PU, and IMN–PU. The characteristic peaks of PUE appear in Figure 1A. The –N=C=O stretching peak at 2270 cm^−1^ is absent, confirming that the isocyanate of the prepolymer has completely reacted with the hydroxyl group of the chain extender, forming the urethane group (Figure S1) [36,37]. The characteristic N–H stretching peak and carbonyl –C=O stretching peak of urethane appear in the regions 3500–3200 and 1733–1700 cm^−1^, respectively. The urethane linkage was confirmed by the –CN peak of carbamate at 1536 cm^−1^ and the amide peak at 1223 cm^−1^ [38,39]. The N–H stretching peaks of the PUEs are compared in Figure 1B. The absence of the free N–H stretching peak at 3480 cm^−1^ clarifies that almost all the N–H groups were hydrogen bonded. The absorbances in the 3300–3350 and 3290–3310 cm^−1^ regions are attributed to N–H groups hydrogen bonded to the carbonyl oxygen of urethane and to the oxygen of ether respectively. The absorbance peak of N–H at a higher wavenumber in IMN–PU than those in BD–PU and ISB–PU indicates stronger bonding of the hard segment in IMN–PU than in BD–PU and ISB–PU. Figure 1C compares the FT-IR spectra of the C=O stretching peaks at 1500–1800 cm^−1^ for the three PUs. Hydrogen-bond formations of the carbonyl groups of PU are well known and reflect microphase separation of the soft and hard segment domains of the PU. Hydrogen bonding shifts the peak of the carbonyl group to lower wavenumbers (ca. 1710 cm^−1^, versus 1730 cm^−1^ for free carbonyl groups). The ratio of the absorbances at 1730 and 1710 cm^−1^ can assay the number of hydrogen bonds in the hard segments of PU. The absorbance ratios of BD–PU, ISB–PU, and IMN–PU were 0.89, 0.81, and 1.18, respectively, verifying that the hard segments of IMN–PU formed a higher proportion of strong hydrogen bonds than those of BD–PU and ISB–PU [40]. The strong hydrogen bonds in IMN–PU are attributable to the conformation of IMN (endo-positioned hydroxyl groups), which favors hydrogen bonding and microphase separation as shown in Figure 1C.

The molecular weights of the PUEs determined by GPC are summarized in Table 1. The molecular weight of BD–PU was the highest among the three PUs, because the secondary hydroxyl groups of ISB and IMN react less readily with the isocyanate group than the primary hydroxyl group of BD [41]. In the ISB- and IMN-based PUs, the final molecular weight depends on the position of the secondary hydroxyl group. IMN–PU has a lower molecular weight than ISB–PU because its reactivity is impeded by the steric hindrance of the endo-hydroxyl groups (the exo-hydroxyl groups are comparatively free) [20].

### 2.2. Thermal and Mechanical Properties of PUEs

**Figure 2:**
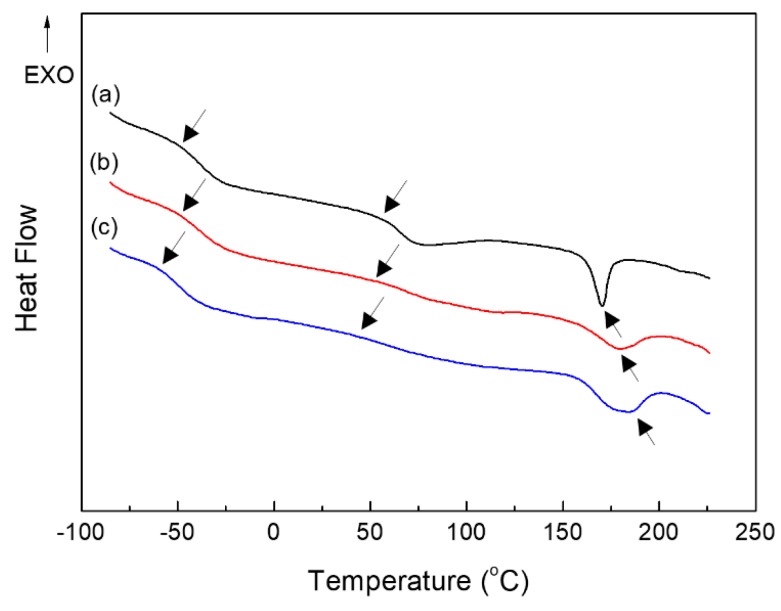
DSC thermograms of PUEs: (a) BD–PU, (b) ISB–PU, and (c) IMN–PU.

The microphase separation of PUEs can be also studied by DSC and DMA. Figure 2 shows the DSC thermograms of the PUEs prepared in this study. The enthalpy changes at low temperatures (−60 to −50°C) are related to glass transitions of the soft segment domain. It is worthwhile noting that the glass transition temperature of the soft segments (*T*_gs_) of IMN–PU is lower than those of BD–PU and ISB–PU. The low *T*_gs_ of polyol in the soft segment of IMN–PU is attributable to better microphase separation than in the other PUEs [42]. The hard segments of the three PUEs melted in similar temperature ranges (150 °C to 200 °C), although the enthalpy change (∆*H*_mh_) of the hard segment melt was larger in IMN–PU than in BD–PU and ISB–PU, again indicating the superior microphase separation, strong hydrogen bonds, and high crystallinity of IMN–PU [43,44]. Glass transition temperatures of hard segments of PUE (*T*_gh_) were observed between *T*_gs_ and *T*_mh_ at 30~60 °C. 

**Figure 3:**
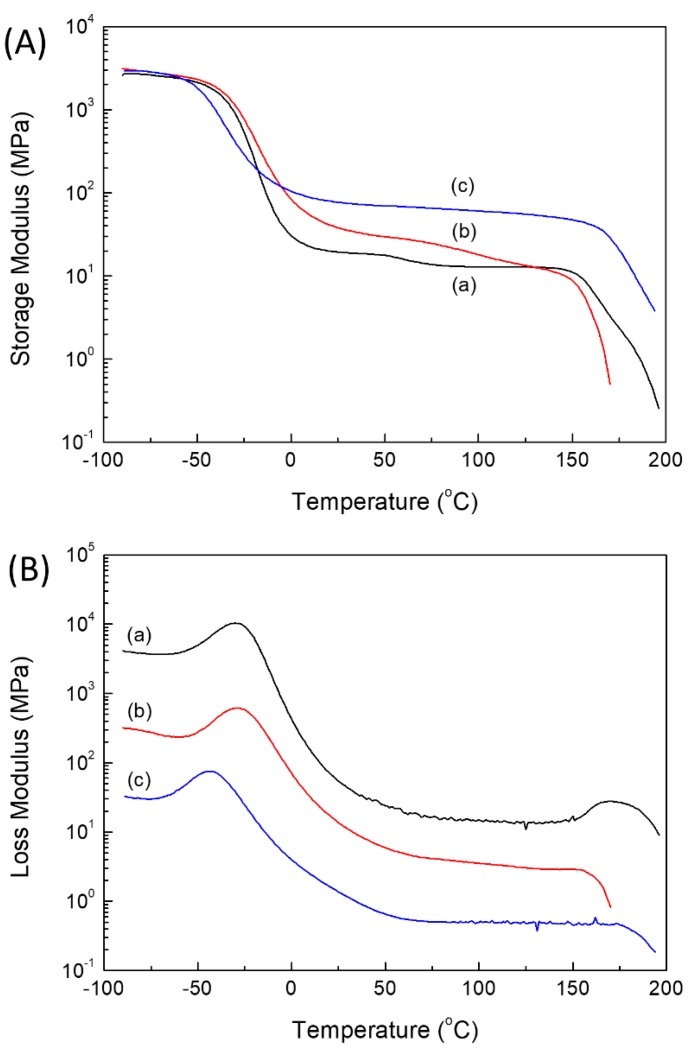
Storage moduli (**A**) and loss moduli (**B**) of (a) BD–PU, (b) ISB–PU, and (c) IMN–PU.

Figure 3 displays the dynamic mechanical properties of the investigated PUEs. The rubbery plateau modulus of IMN–PU was higher than those of BD–PU and ISB–PU in (A) of Figure 3. This high rubbery plateau modulus reflects the structural features and physical crosslinking effects of the hard segment domain. The flow temperature (*T*_flow_) defined as the intersection of the rubbery plateau region and flow region was also higher in IMN–PU than in the other PUs. The stronger the hydrogen bond of PUEs, the higher the flow temperature of PUEs. Figure 3B compares the loss moduli in the three PUEs. *T*_gs_ (indicated by the temperature of the highest loss modulus) was lower in IMN–PU than in BD–PU and ISB–PU. Again, this reflects the better microphase separation in IMN–PU. The thermal properties determined by DSC and DMA are summarized in Table 2.

**Figure 4:**
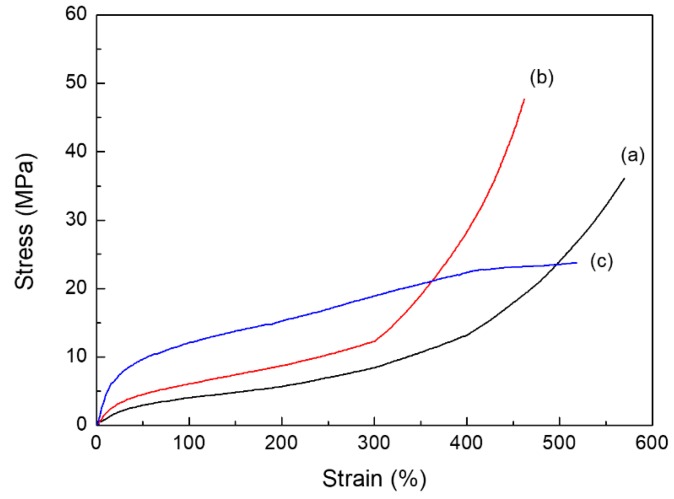
Stress–strain curves of (a) BD–PU, (b) ISB–PU, and (c) IMN–PU.

Figure 4 shows the stress–strain behaviors of the PUs. The tensile properties of PUEs determined by UTM are summarized in Table 3. The Young’s moduli of ISB–PU and IMN–PU were higher than that of BD–PU, owing to the rigid structure of the anhydrohexitols [45]. Interestingly, ISB–PU and BD–PU showed strain hardening (i.e., high tensile strength), whereas IMN–PU did not due to strong hydrogen bonds. According to Torkelson and coworkers, strain hardening in PUs with high microphase separation is hindered by the sharp boundary between the microphases [46]. Efficient strain hardening of ISB–PU is attributable to the interdiffusion of the interfacial properties of the microphase-separated domains.

### 2.3. Morphologies of PUEs

**Figure 5:**
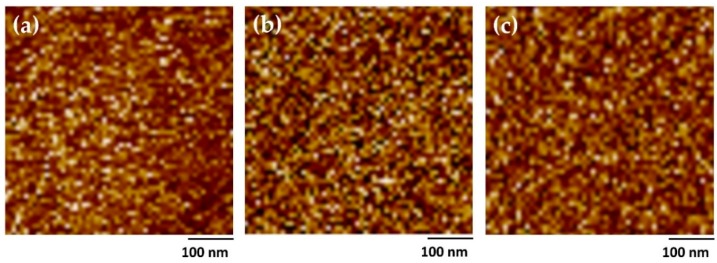
AFM images of microphase separated PUEs obtained in tapping mode: (**a**) BD–PU; (**b**) ISB–PU; (**c**) IMN–PU.

The microstructure and phase separation of the PUEs were confirmed by tapping-mode AFM and SAXS. Figure 5 displays AFM images of the PUEs. The darker and brighter regions correspond to the soft and hard segment domains, respectively [47,48]. In BD–PU (Figure 5a), the bright regions of sphere or cylinder like shapes are isolated and surrounded by the dark regions. This apparent phase difference evidences the contrast between the hard and soft segment domains. In ISB–PU and IMN-PU, the bright portions are smaller than in BD–PU slightly, but the striking differences between PUEs in this study were not observed. 

**Figure 6:**
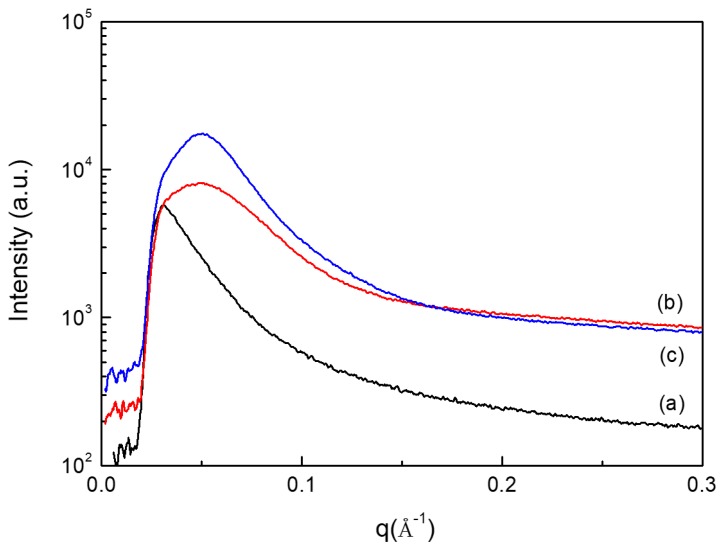
SAXS profiles of (a) BD–PU, (b) ISB–PU, and (c) IMN–PU.

Figure 6 shows the SAXS profiles of the PUEs investigated in this study. In the SAXS profile, the scattering intensity indicates crystallinity, which was related to the microphase separation of PUEs [45,49,50]. The scattering intensities of IMN–PU and ISB-PU were higher that of BD–PU, which contributed to the chemical structure of anhydrohexitol. The interdomain distances of PUEs were calculated by Bragg’s equation, d = 2π/q, using the value of q at the peak. The scattering widths of IMN–PU and ISB–PU were bigger than that of BD–PU, which indicate that the sizes of hard domain in IMN–PU and ISB–PU were smaller than that of BD–PU. On the other hand, the calculated average interdomain distances of BD–PU, IMD–PU and ISB–PU were 20 nm, 12 nm and 12 nm, respectively, indicating better microphase separation of BD–PU. These results showed that strong hard segment bonding of IMN–PU despite the small size of domain. This is consistent with the previous results of DSC, DMA and AFM.

### 2.4. Reversibility of PUEs

**Figure 7:**
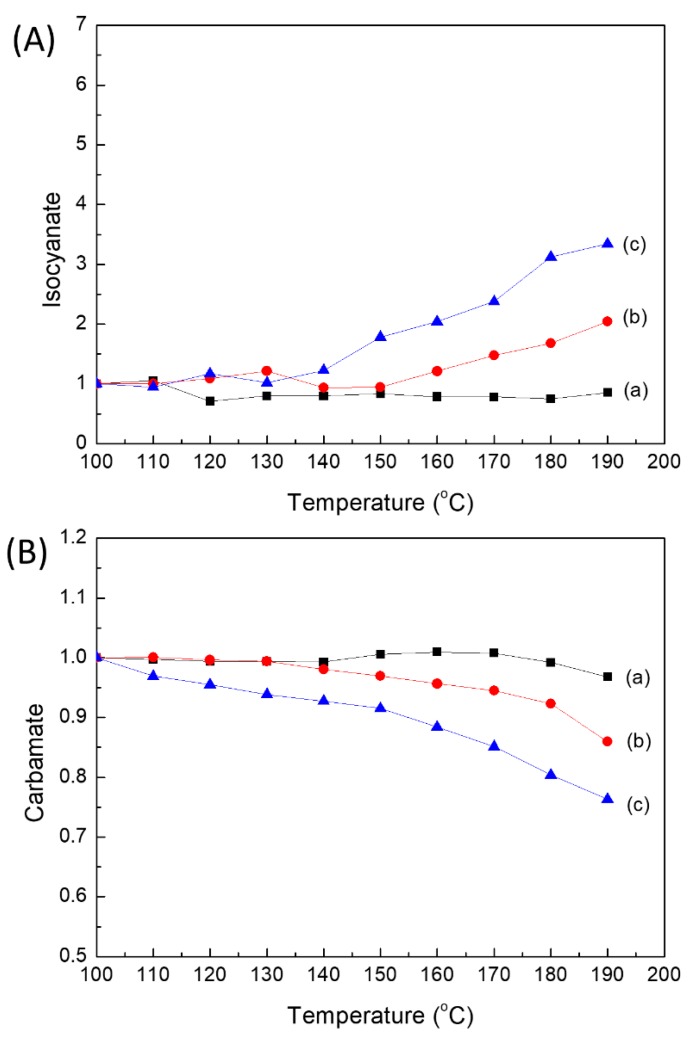
Temperature dependences of the relative intensities of the isocyanate peak at 2270 cm^−1^ (**A**) and carbamate peak at 1536 cm^−1^ (**B**) in FT-IR spectra: (a) BD–PU, (b) ISB–PU, and (c) IMN–PU.

**Scheme 2:**
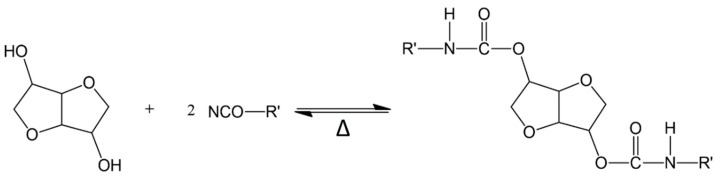
Thermoreversible reaction of anhydrohexitol-based urethane.

ISB–PU and IMN–PU contains urethane bonds from anhydrohexitol and MDI (an aromatic isocyanate). As is well known, urethane bonds formed by aromatic hydroxyl groups and aromatic isocyanates undergo reversible reactions at elevated temperatures [51]. Such reversibility is used to prepare blocked isocyanates. Anhydrohexitol possesses a hydroxyl group with a cycloaliphatic ring structure similar to the aromatic hydroxyl group, and a very bulky structure. For this reason, we were interested in the reversible features of anhydrohexitol-based urethanes for the self-healing PUEs. The reversible properties of the carbamates in ISB–PU and IMN–PU were investigated by FT-IR at elevated temperatures. The FT-IR spectra of ISB–PU and IMN–PU exhibited an –N=C=O peak at elevated temperatures (Figure S2), confirming the reversible feature of these PUs. In contrast, the –N=C=O peak was absent in the BD–PU spectrum at the same temperatures. Figure 7 plots the relative absorbances of the isocyanates and the carbamate C–N group in the three PUs as a function of temperature. The peak intensities of ISB–PU and IMN–PU in Figure 7A increased with temperature, whereas those of BD–PU were almost constant. The significant decline in the carbamate C–N peaks of ISB–PU and IMN–PU in Figure 7B was attributable to the reverse reaction of urethane. Moreover, as the reversible reaction progressed, the increase in the isocyanate peak and the corresponding decrease in the carbamate peak were greater in IMN–PU than in ISB–PU. The urethane group formed by the hydroxyl group at the endo-position of anhydrohexitol is destabilized by the steric hindrance caused by intramolecular hydrogen bonding. These unstable urethanes favor reversible reactions as shown in Scheme 2.

**Figure 8:**
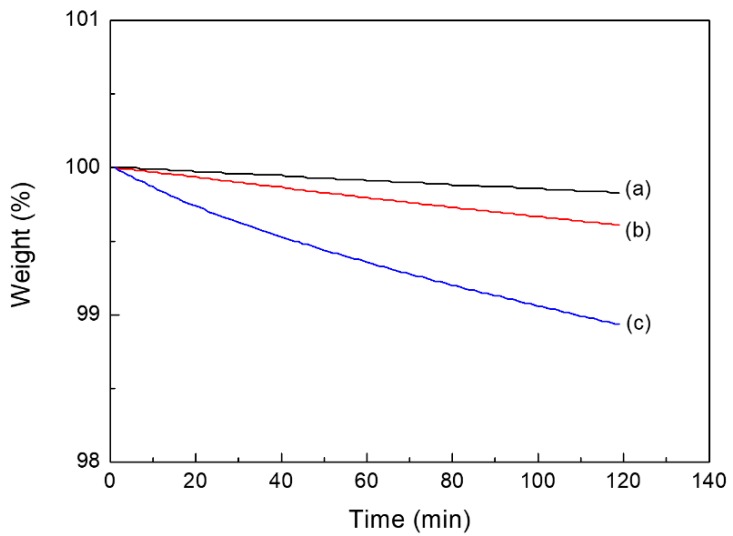
Isothermal TGA weight loss curves of PUEs at 180 °C: (a) BD–PU, (b) ISB–PU, and (c) IMN–PU.

To check whether volatile anhydrohexitols are liberated by the reversible properties of carbamates in the PUs, we carried out an isothermal TGA at 180 °C on very thin PUE films. In isothermal runs, the weight loss is attributable to the volatilizations of ISB and IMN dissociated by reversible reaction of urethanes. These results are due to the isothermal condition exceed the boiling points of both ISB and IMN (150 °C and 160 °C, respectively). Figure 8 plots the weight losses at 180 °C in BD–PU, ISB–PU, and IMN–PU over time. After 120 min, the weight of BD–PU was only marginally reduced, whereas those of ISB–PU and IMN–PU had noticeably declined. We thus postulated that the carbamates of ISB–PU and IMN–PU chemically reverse at lower temperature than those of BD–PU, conventional PU comparatively. TGA data of PUEs are given in Figure S3. The initial weight loss of ISB-PU and IMN-PU was observed at lower temperatures than that of BD-PU due to the reversible features anhydrohexitol-based PU as discussed above.

### 2.5. Reprocessability of ISB-Based PU

**Figure 9:**
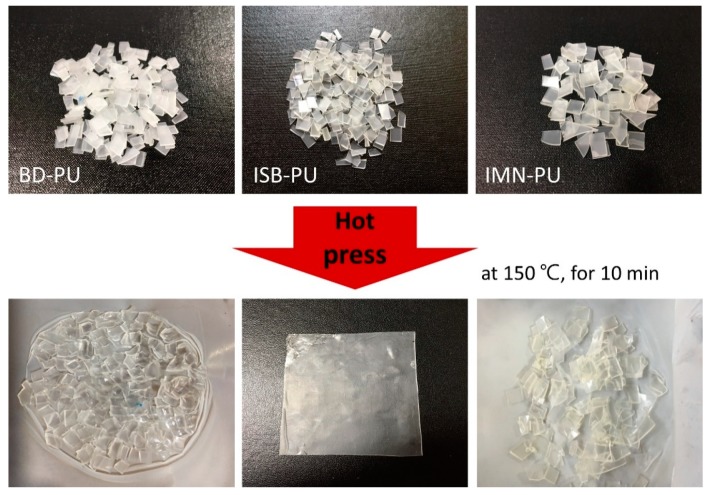
Reprocessing images of PUE test pieces before (**up**) and after (**bottom**) 10 min at 150 °C.

The reprocessing tests demonstrate whether ISB-based PU exhibits self-healing properties. When the ISB–PU film is damaged, the fractured pieces can be reprocessed into integrated film via the thermoreversible behavior of the PU. In the reprocessing analysis, the ISB–PU and BD–PU test pieces were placed between two metal plates and hot pressed at 150 °C for 10 min. Photo images of fractured BD–PU and ISB–PU films before and after heating are shown in Figure 9. After remolding, the ISB–PU film was reintegrated through reversible covalent bonding and physical melting. In contrast, the BD–PU test pieces remained nonintegrated and adhered only through partial melting, retaining a fractured appearance. IMN–PU also did not recover its shape after hot pressing. IMN-PU exhibited high reversibility than ISB–PU, but the flow temperature of IMN-PU was increased by the improved microphase separation.

## 3. Conclusions

We have synthesized PUEs with biobased isosorbide and isomannide as chain extenders and compared their properties with those of PUE synthesized with a BD chain extender. The anhydrohexitol-extended PUEs exhibited higher mechanical properties than the BD-extended PUE. The morphologies of the synthesized PUEs depends on the chemical structure and microphase separations of hard segments composed of chain extender molecules. As confirmed by the FT-IR spectra and TGA results, the anhydrohexitol-based PUs exhibited thermal reversibility with their constituents; moreover, a fractured ISB–PU film reformed after hot pressing at 150 °C. It is inferred that the anhydrohexitols due to the rigid, bulky structure of ISB and IMN as chain extenders conferred more thermal reversibility to PUE than BD, the conventional chain extender. It was found that ISB-PU showed superior mechanical properties compared with BD-PU and IMN-PU. Owing to its reversibility and excellent mechanical properties, ISB-based PU is a promising candidate for hot melt adhesives, powder coatings, and self-healing polymers.

## 4. Materials and Methods

### 4.1. Materials

The PU prepolymer was prepared from poly(tetramethylene ether glycol) (PTMEG; Aldrich, Young-in, Korea) with a number-average molecular weight of 1000 g/mol as the polyol and 4,4′-methylenebis(phenyl isocyanate) (MDI; Aldrich) as the diisocyanate. The chain extenders were 1,4-butanediol (BD; Aldrich), isosorbide (ISB; Samyang Corporation, Dae-jeon, Korea), and isomannide (IMN; Aldrich). Prior to the reaction, the polyol was dehydrated under vacuum conditions at 60 °C for 1 day, and ISB and IMN were dried in a vacuum evaporator at 90 °C for 6 h.

### 4.2. Synthesis of PUEs

PUEs were prepared by a two-step polymerization method. The prescriptions of the materials are shown in Table 1. In the first step, prepolymer was prepared from the polyol and excess diisocyanate, forming an NCO-terminated molecule by the reactions of PTMEG and MDI at 60 °C under nitrogen gas. The hydroxyl group of the polyol end was reacted with isocyanate until the NCO content of the prepolymer reached the theoretical NCO content, determined by the *n*-dibutylamine back-titration method in ASTM 2572-97. In the second step, the prepolymer was extended by adding BD, ISB, or IMN to the prepared prepolymer with rapid stirring. After removing the bubbles, the mixture of prepolymer and chain extender was cast into a mold and cured in an oven at 110 °C for 24 h. Completion of the reaction was confirmed by the disappearance of the isocyanate peak (2270 cm^−1^) in the Fourier transform infrared (FT-IR) spectrum.

### 4.3. Characterization

To confirm PUE synthesis and identify the isocyanate group released by the reversible reaction of urethane at elevated temperatures (100–200 °C), the PUEs were analyzed by FT-IR spectroscopy (FT-IR-302, Jasco, Tokyo, Japan). The average molecular weights of the PUEs were measured by gel permeation chromatography (GPC; Agilent 1200s, Palo Alto, CA, USA) with RI detectors (Wyatt, Optilab rEX, Santa Barbara, CA, USA). A dimethylformamide/tetrahydrofuran (1/1 weight ratio) mixture was used to dissolve samples. The calibration was performed using polystyrene standards. The thermal properties were investigated using a differential scanning calorimeter (DSC; Q20, TA Instruments, New Castle, DE, USA) heated from −90 °C to 220 °C at 10 °C/min under a nitrogen atmosphere. The dynamic mechanical properties of PUEs were characterized using a dynamic mechanical analyzer (DMA; Q800, TA Instruments) heated from −90 °C to 200 °C at 5 °C/min in film tension mode. All samples were tested under a nitrogen atmosphere. The tensile properties of the PUEs were measured with a universal testing machine (UTM; LR5K, LLOYD, West Sussex, UK). The tensile test was carried out by pulling the dog-bone-shaped samples at 500 mm/min at room temperature, following ASTM D638 (Type IV). Three specimens per sample were measured to get the average value. The morphologies of PUEs were confirmed by an atomic force microscope (AFM; Multimode-8, Bruker, Billerica, MA, USA) in tapping mode with a nanoscope V (Bruker) controller. The AFM samples were prepared by casting 5 wt% of the PUEs in DMF solvent on a glass slide, followed by drying at 80 °C for 1 day in an oven. The microphase separations of PUEs were investigated employing a small angle X-ray scattering instrument (SAXS; D8 discover, Bruker, Walpole, MA, USA). Thermal gravimetric analysis of PUEs was performed by thermogravimetric analyzer (TGA; Q50, TA Instruments). The thin PUE samples were rapidly heated from room temperature to 180 °C and maintained an isothermal condition under a nitrogen atmosphere for 120 min. Also, TGA curves of PUEs were obtained by heating from room temperature to 800° C at 20 °C/min in nitrogen gas environment.

## Figures and Tables

**Table 1:** Composition and average molecular weight of PUEs weight determined by GPC.

Sample Code	Composition (Molar Ratio)					Average Molecular Weight		
	Prepolymer		Chain Extender			Mn	Mw	Ð
	PTMEG	MDI	BD	ISB	IMN	(g/mol)	(g/mol)	
BD–PU	1	2	1	-	-	27,000	45,000	1.67
ISB–PU	1	2	-	1	-	23,000	44,000	1.91
IMN–PU	1	2	-	-	1	16,000	34,000	2.12

**Table 2:** Thermal properties of PUEs.

Sample Code	DSC				DMA	
	*T*_gs_ (°C)	*T*_gh_ (°C)	*T*_mh_ (°C)	Δ*H*_mh_ (J/g)	*T*_gs_ (°C)	*T*_flow_ (°C)
BD–PU	−50	58	151–183	4.7	−30	151
ISB–PU	−51	57	147–198	4.6	−29	145
IMN–PU	−60	47	151–199	7.2	−44	168

**Table 3:** Tensile properties of PUEs.

Sample Code	Young’s Modulus (MPa)	Tensile Strength (MPa)	Elongation at Break (%)
BD–PU	8 (±0.3)	36 (±2.1)	570 (±30.1)
ISB–PU	14 (±0.8)	48 (±3.4)	462 (±49.0)
IMN–PU	39 (±1.2)	24 (±2.1))	518 (±21.1)

## Supplementary Materials

The following are available online, Figure S1. FT-IR spectra of raw materials (A), synthesized prepolymer and PUEs (B): (a) PTMEG, (b) MDI, (c) BD, (d) ISB, (e) IMN, (f) prepolymer, (g) BD-PU, (h) ISB-PU, (i) IMN-PU, Figure S2. FT-IR spectra of PUEs at elevated temperatures: (a) BD–PU, (b) ISB–PU, and (c) IMN–PU. In FT-IR spectra of ISB-PU and IMN-PU, the peak intensities of absorbances due to isocyanate groups generated by the reversible urethane bods increased with heating while those of BD-PU did hardly, Figure S3. TGA thermograms and 1st derivative of TGA curve of PUEs: (a) BD–PU, (b) ISB–PU, and (c) IMN–PU.
