# Supplementary material for: Preparation and Characterization of Isosorbide-Based Self-Healable Polyurethane Elastomers with Thermally Reversible Bonds
Source: Molecules. 2019 Mar 18;24(6):1061. doi: 10.3390/molecules24061061 (PMC6471067; doi:10.3390/molecules24061061)
Supplement: Supplementary file 1 [file molecules-24-01061-s001.pdf]

< Supplementary Information >

Preparation and characterization of isosorbide-based self-healable polyurethane elastomers with thermally reversible bonds

Han-Na Kim [1,†], Dae-Woo Lee [1,†], Hoon Ryu [2], Gwang-Seok Song [2], and Dai-Soo Lee [1,*]

[1] Division of Semiconductor and Chemical Engineering, Chonbuk National University, 567 Baekjedaero , Deokjin-gu, Jeonju, 54896, Republic of Korea; hnk07@hanmail.net (H.N.K.); dwlee2310@hanmail.net (D.W.L.)

[2] Industrial Biotechnology Program, Chemical R&D Center, Samyang Corporation, Daedeok-daero 730, Yuseong-gu, Daejeon 34055, Republic of Korea; hoon.ryu@samyang.com (H.R); gwangseok.song@samyang.com (G.S.S)

† Contributed equally to this work

* Correspondence: daisoolee@jbnu.ac.kr (D.S.L); Tel.: +82-63-270-2310

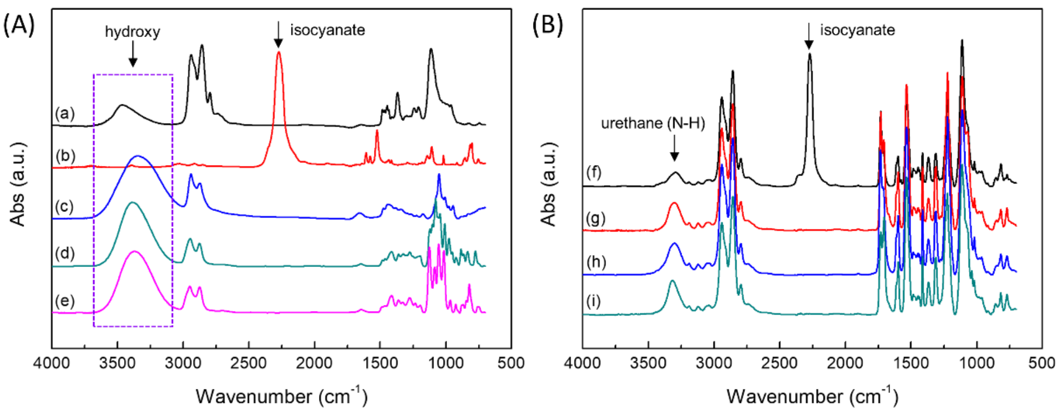

Figure S1. FT-IR spectra of raw materials(A), synthesized prepolymer and PUEs(B): (a) PTMEG; (b) MDI; (c) BD; (d) ISB; (e) IMN; (f) prepolymer; (g) BD-PU; (h) ISB-PU; (i) IMN-PU.

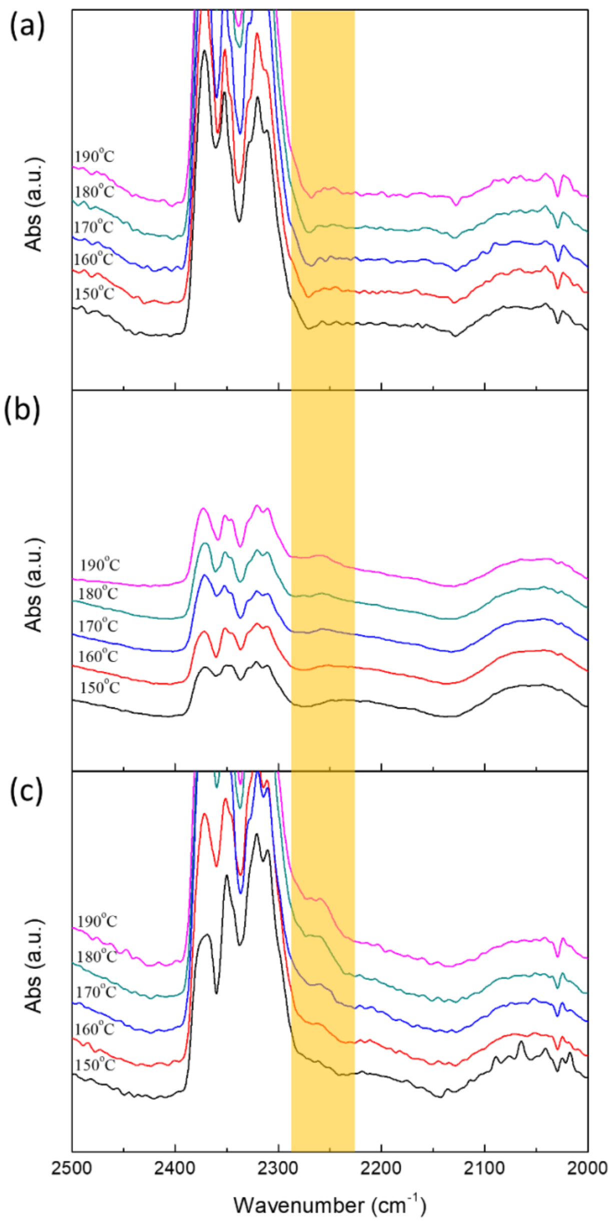

Figure S2. FT-IR spectra of PUEs at elevated temperatures: (a) BD–PU, (b) ISB–PU, and (c) IMN–PU. In FT-IR spectra of ISB-PU and IMN-PU, the peak intensities of absorbances due to isocyanate groups generated by the reversible urethane bods increased with heating while those of BD-PU did hardly.

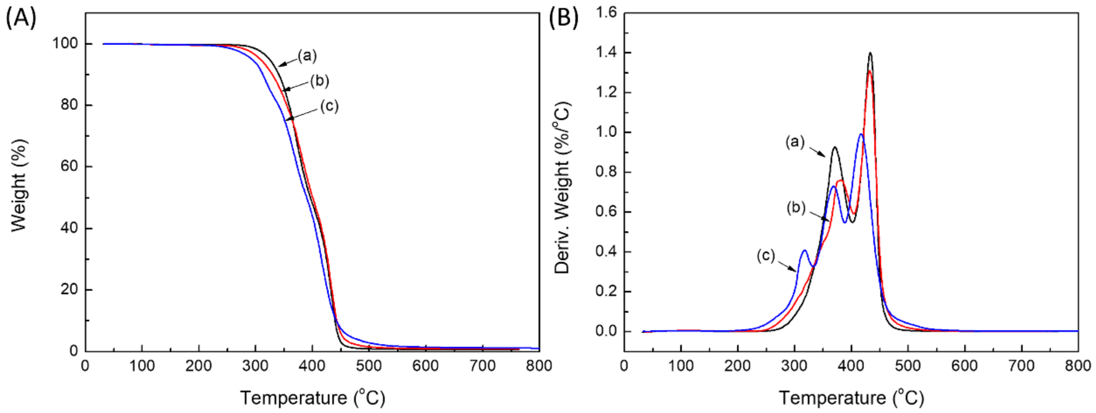

Figure S3. TGA thermograms and 1st derivative of TGA curve of PUEs: (a) BD–PU, (b) ISB–PU, and (c) IMN–PU.
